# Unmet Family Planning Need: Differences and Levels of Agreement Between Husband-Wife, Haryana, India

**DOI:** 10.4103/0970-0218.55281

**Published:** 2009-07

**Authors:** Kapil Yadav, Bir Singh, Kiran Goswami

**Affiliations:** Centre for Community Medicine, All India Institute of Medical Sciences, New Delhi, India

**Keywords:** Agreement, family planning, Kappa statistics, unmet need of family planning

## Abstract

**Research question:**

Is there agreement between husbands and wives regarding unmet need of family planning?

**Hypothesis:**

The unmet need of family planning is perceived more by women then their husbands.

**Objective:**

1) To ascertain the unmet needs of family planning for husbands and wives. 2) To ascertain the level of agreement between husbands and wives regarding unmet needs of family planning.

**Design:**

A cross-sectional survey

**Setting:**

Dayalpur village in Intensive field practice area of Comprehensive Rural Health Services project (CRHSP), Ballabgarh, Haryana.

**Study Period:**

July 2003- June 2005.

**Participants:**

included 200 married couples selected by simple random sampling.

**Statistical Analysis:**

Level of agreement between husbands and wives was analyzed using Kappa statistics.

**Results:**

Unmet need for family planning was 11% (22 out of 200) for husbands and 17.5% (35 out of 200) for wives. The difference was seen both in unmet need for spacing (M-3.5% vs. F-6%) as well as limiting family size (M-7.5% vs. F-11.5%). Overall, 93.5% concordance was observed amongst husbands and wives. In all the cases where disagreement was seen (6.5%), wives reported having unmet need for contraception whereas their husbands perceived none. The unadjusted Kappa statistic was 0.73 and prevalence adjusted Kappa was 0.88.

**Conclusion:**

Unmet need for family planning was significantly higher for wives compared to husbands. Despite high degrees of agreement amongst the couples, the nature of disconcordance reinforces the need for policy makers to take into account the perspective of men.

## Introduction

India was the first nation in the world to start a family planning programme at the national level, in 1951. Over the years, the crude birth rate of India has declined from 40.8 (1951) to 26.4 (1999 SRS), infant mortality rate declined from 146 per 1000 live births (1951) to 72 per 1000 live births (1998 SRS).(1)

However, one criticism that has often been reiterated for the family welfare programme of India is that it has not become a people's own programme. The family welfare programme focused purely on demographic goals and concentrated on numerical, method-specific contraceptive targets till the advent of the “target free approach”.(2) Female sterilization accounted for three-quarters of the modern methods of contraception used in India, only 3.4% of couples rely on vasectomy and 2.4% rely on condoms.(3) This is because, while the terminal methods, particularly female sterilization have been promoted consciously, participation of men lagged behind. This lack of involvement of men in family planning has attracted attention since early eighties but has become a focus of attention during the last decade, particularly after the International Conference on Population and development (ICPD), Cairo (1994) and the World Conference on Women at Beijing (1995).(4,5)

New perspectives on men have emerged from an evolution in thinking about reproductive health with ICPD programme of action laying down a holistic concept of reproductive health. With the expansion of the definition of reproductive health to include “… right of men and women to be informed and to have access to safe, effective, affordable and acceptable methods of family planning of their choice…” following ICPD (1994), focus for most of the reproductive health component shifted has from women to couples.(6) An important concept in family planning is addressing the male ‘KAP-Gap’.

‘KAP-Gap’ or ‘Unmet-Need of family planning’ is defined as the difference between fertility preferences and current fertility behavior.(7) About 201 million women still have an unmet need for effective contraception(8) Meeting the unmet need of family planning is one of the immediate objectives of the National Population policy (2000) of India.(9) Researchers have questioned the validity of the es-timates of the unmet need derived from information collected only from women.(10,11) Any calculation of unmet need that fails to take into account the desires of male partners may not be valid for estimating future levels of contraceptive adoption. Imputing male intentions may allow a clearer understanding of why family planning programs have not been more successful in developing countries. This study was designed to assess the unmet need of family planning for husbands and wives and ascertain the level of agreement amongst them.

## Materials and Methods

### Study area and population:

The study was conducted in Dayalpur Village, Ballabgarh block of district Faridabad, Haryana, India. It is approximately 40 kilometres South-West of New Delhi. Dayalpur is one of the 28 villages under the Comprehensive Rural Health Services Project (CRHSP) Ballabgarh, the rural field practice area of Centre for Community Medicine (CCM), All India Institute of Medical Sciences, New Delhi. It has a 60-bedded secondary level hospital and two Primary Health Centre (PHC) at villages Dayalpur and Chhainsa. Community-based preventive and promotive services are provided to the population under the two PHCs (total population approx. 80,000 in 2003).The total population of Dayalpur village was about 6400 in July, 2003 (Study year). The reference population of the study consisted of couples residing in Dayalpur village. The study population included married women aged 15-44 years living with their husbands. Married women were used as the entry point. The study unit included a couple; analysis was based on couple variables.

### Study design and sample size:

Study design was cross sectional. Due to a time constraint, and limited resources, it was decided to limit the sample size to 200 couples.

### Sampling technique:

The eligible couple list of Dayalpur village was obtained from the Management Information system of C.R.H.S.P Ballabgarh, and was used as sampling frame. A required number of couples were selected using random number table generated using Fox Pro software. The list provided names of the couples and their house number.

### Data collection and statistical analysis:

Data was collected using a pre-tested, semi-structured interview schedule, modified from NFHS-2 questionnaire and was administered by the first author himself. It had two parts. The first part covered socio-economic and demographic information of the respondents. The second part contained information on family planning covering major parts of the research objectives. Most of the questions were pre-coded. The instrument for husbands was similar to that for the wives. It was translated to local language and back translated and pre-tested twenty individuals (ten couples) in the non study area (village Chhainsa). The instrument was finalized after necessary corrections.

The interview was conducted at the respondents' home. Both husband and wife were interviewed by the same interviewer on the same day separately. Since most of the husbands were at work during working hours, their interviews were conducted in evenings. None of the couples approached for interview refused to give consent to participate. Data was entered in EPI-Info, 2002. Data analysis was performed with Microsoft Excel (Windows Office 2000) and SPSS (Version 10 for windows). Proportions, mean, standard deviation, median and range were used for descriptive statistics. Level of agreement amongst spouses/partners was analyzed using Kappa statistics. As Kappa statistics may be influenced by high prevalence of outcome under consideration, prevalence Adjusted Kappa was used wherever appropriate.(12)

## Results

### Socio-demographic characteristics:

Most of the husbands were aged between 25 to39 years whereas wives were between 20 to 34 years. The mean age was observed to be higher for husbands (33.4 plus/minus 7.3 yrs) then wives (28.5 plus/minus 6.5 yrs). Compared to husbands, proportionally, more wives were less than 25 years old (31% vs. 7%) while greater percentage of husbands were in age groups above 35 years (42% vs. 21%). Illiteracy was reported more by wives (32.5%) as compared to husbands (9%). Education higher than high school was reported by 71.5% of husbands and 41% of wives. The mean duration of schooling for husbands was 10.1 years (SD- 4 years) compared to 6.5 years (SD-5.1 years) of wives. Majority of the study population belonged to lower-middle class (56.5%), followed by middle class (35%), in lower class (4.5%), upper-middle class (3.5%) and upper class (.5%).

### Unmet need of family planning in husbands and wives:

Only 11% of husbands reported having unmet need of family planning as compared to 17.5% of wives, and the difference was highly significant statistically (*P* value<.001) [Table 1]. This overall difference was maintained if the data was analyzed separately for spacing and limiting only. About 3.5% of husbands reported having unmet need for spacing compared to six per cent of wives. Similarly, 7.5% of husbands compared to 11.5% of wives reported unmet need for limiting. Of the 11 couples with pregnant/lactating, none of the husbands reported that the pregnancy was unwanted or mistimed while five wives reported so [Table 1]. None of the earlier studies have documented similar findings and the possible explanation could be dominance of males in deciding pregnancy or males being less forthcoming then females on issue of unwanted pregnancy.

**Table 1:** Agreement between husband-wife on unmet need of family planning

Unmet need for family planning		Wives		Total
			
		No	Yes	
Husbands	No	165 (82.5)	13 (6.5)	178 (89.0)
	Yes	0 (0)	22 (11)	22 (11)
Total		178 (89)	35 (17.5)	200 (100)

* n (percentage)

### Husband-wife agreement on unmet need for family planning:

Concordance of 93.5% was observed amongst husbands and wives regarding unmet need of family planning. In majority of the cases (82.5%) both husbands and wives did not have unmet need of family planning. In 6.5% cases both had unmet need. The unadjusted kappa statistic was 0.73 and prevalence adjusted kappa was 0.88. In all cases where disagreement was seen (6.5%), wives reported having unmet need for contraception whereas their husbands had none. There were no cases when husband reported unmet need and wife did not. This finding indirectly reflects the possibility that it is husbands who have a predominant role in deciding use of contraception and even though wives may not want more children, they cannot use contraception without their husbands consent. Thus, estimates of unmet need of family planning, based on wives perception and responses alone, (as is routine practise in various national DHS surveys) may be over-estimates and there is a need for formulating policies keeping husbands perspective in view.

## Discussion

Unmet need for family planning was higher for wives compared to husbands (11% vs 17.5%, *P* <.001) in study respondents. The difference was seen both in unmet need for spacing (H-3.5% vs. W-6%) as well as for limiting family size (H-7.5% vs. W-11.5%). Overall concordance 93.5% and agreement (prevalence adjusted Kappa was 0.88) between husbands and wives for unmet need, though very good, was significantly low compared to perfect agreement.

We have concurrently looked at the unmet needs of husbands and wives, unlike previous studies, thus possibly capturing the difference in a more valid way. The fact that interviews were conducted by a male researcher may affect the internal validity of study especially responses of wives. However, every effort was made to offset this by establishing good rapport with respondents. The results of the study can be generalised only to population of Dayalpur village or to the rural field practise area of CRHSP. Any generalisation to a wider population should be done with caution at it may not share similar socio-demographic profile as our study population.

The results of our study are similar to NFHS II data reporting 7.6% unmet need for family planning, with unmet need for spacing being 2.9% and same for limiting family being 4.7%.(8) However, a higher unmet need has been observed in present study which may be due to the higher literacy level of respondents leading to higher awareness. Similar results were reported in a study from Agra, 29% of husbands compared to 39% of wives reported having unmet need for family planning.(10) However, unlike our study, the difference observed was predominantly seen due to difference in unmet need of spacing.

None of the previous studies have looked at agreement between husbands and wives on the unmet need for family planning. However, several authors have looked at agreement regarding current contraception use and fertility desire, the two variables that combine to make unmet need for family planning. The findings in these studies were similar to those observed in our study i.e. high degree of agreement and females reporting greater unmet need of family planning.(12)

The fact that our study sample was restricted because of logistic constraints may have affected the precision of our estimates of unmet need. But the difference between husband-wife's unmet need is still valid as it is statistically significant even with the current sample size. The findings of this study, though internally valid, cannot be generalised to larger population.

The findings of our study have highlighted the significant difference in husband-wife's unmet need of family planning and re-enforce the fact that responses of wives alone may not suffice The policy makers should take into account the desires of both to target the unmet need and other family planning issues.

## Conclusion

There is significant difference between husband and wife's unmet need for family planning. This needs to be factored in family welfare planning and policy making as estimation from only wives responses might not be reliable.
